# Identification and Validation of a Novel Immune-Related Four-lncRNA Signature for Lung Adenocarcinoma

**DOI:** 10.3389/fgene.2021.639254

**Published:** 2021-02-23

**Authors:** Jixin Wang, Xiangjun Yin, Yin-Qiang Zhang, Xuming Ji

**Affiliations:** ^1^Zhejiang University-University of Edinburgh Institute, Zhejiang University, Zhejiang, China; ^2^School of Basic Medical Science, Zhejiang Chinese Medical University, Zhejiang, China; ^3^Department of Hepatic Diseases, Xiyuan Hospital, China Academy of Chinese Medical Sciences, Beijing, China

**Keywords:** lung adenocarcinoma, lncRNA, survival analysis, immune infiltrate, GSEA

## Abstract

Lung adenocarcinoma (LUAD) is a major subtype of lung cancer, the prognosis of patients with which is associated with both lncRNAs and cancer immunity. In this study, we collected gene expression data of 585 LUAD patients from The Cancer Genome Atlas (TCGA) database and 605 subjects from the Gene Expression Omnibus (GEO) database. LUAD patients were divided into high and low immune-cell-infiltrated groups according to the single sample gene set enrichment analysis (ssGSEA) algorithm to identify differentially expressed genes (DEGs). Based on the 49 immune-related DE lncRNAs, a four-lncRNA prognostic signature was constructed by applying least absolute shrinkage and selection operator (LASSO) regression, univariate Cox regression, and stepwise multivariate Cox regression in sequence. Kaplan–Meier curve, ROC analysis, and the testing GEO datasets verified the effectiveness of the signature in predicting overall survival (OS). Univariate Cox regression and multivariate Cox regression suggested that the signature was an independent prognostic factor. The correlation analysis revealed that the infiltration immune cell subtypes were related to these lncRNAs.

## Introduction

Lung cancer is one of the most common types of malignancy that is a leading cause of death worldwide. The frequency of lung adenocarcinoma (LUAD) has exceeded lung squamous cell carcinoma (LUSC), which makes LUAD the most common histological subtype of primary lung cancer (Lortet-Tieulent et al., 2014). The high mortality is mainly because lung cancer is typically diagnosed at an advanced stage. Patients who have mutations in epidermal growth factor receptor (EGFR) are recommended to receive molecule-targeted therapy by administrating anti-EGFR inhibitors (Duffy and O’Byrne, 2018). For those who do not have specific mutations, immunotherapies targeting inhibitory receptors have recently emerged as an effective therapy for advanced cancer. The most studied way is using antibodies to block the programmed cell death-1 (PD-1)/programmed cell death ligand-1 (PD-L1) pathway, an immune checkpoint that is exploited by tumor cells (Sacher and Gandhi, 2016). These anti-PD-1/PD-L1 treatments do not need specific mutations such as EGFR, KRAS, or ALK (Brody et al., 2017) and are available for more patients.

A large proportion of tumor cells are immune infiltrating cells (Yu et al., 2018). Tumor immune cell infiltration is vital for the effect of immunotherapy and therefore the prognosis of LUAD patients because the tumor-specific antigens need to be recognized by the antigen–antibody complementary determining region in immune cells (Sela-Culang et al., 2014; Jiang et al., 2017; Liu et al., 2018). Higher CD8-T cell infiltration seems to better respond to anti-PD-1/PD-L1 administration (Pagès et al., 2016).

Long non-coding RNA (lncRNA), a type of non-coding RNA with a length longer than 200 nucleotides, accounts for a large proportion of the human genome. Studies have suggested that lncRNAs regulate gene expression and are associated with many biological processes such as development (Quinn and Chang, 2016). For example, PTTG3P up-regulation has been discovered to promote cell viability and contribute to the poor survival of LUAD patients (Shih et al., 2020). Moreover, lncRNA is related to many aspects of cancer immunity including the recognition and killing of cancer cells, cell migration, and T cell infiltration (Yu et al., 2018).

Therefore, it is reasonable to predict the survival of LUAD patients and guide clinical treatment using immune-related lncRNAs. To further explore the possible roles of immune-related lncRNAs that play in prognosis and immunotherapy, we analyzed the transcriptome data from The Cancer Genome Atlas (TCGA) and Gene Expression Omnibus (GEO) database to build an immune-related lncRNA signature.

## Materials and Methods

### LUAD Data Collection and Grouping

TCGA-LUAD datasets including RNA expression profile (*n* = 585) processed by HTcount, patients survival (*n* = 738), and phenotype (*n* = 125) information were downloaded from TCGA^1^. RNA expression profiles of GSE19188 (normal: 65; tumor: 45), GSE27262 (normal: 25; tumor: 25), GSE30219 (normal: 14; tumor: 84), and GSE31210 (normal: 20; tumor: 226) were downloaded from https://www.ncbi.nlm.nih.gov/ and each was normalized by RMA algorithm using R package *affy*. Tumor and normal tissue samples were selected from the above GEO datasets and divided into tumor (*n* = 375) and normal (*n* = 124) groups. The survival data of LUAD patients in GSE30219 (*n* = 84), GSE31210 (*n* = 226), and GSE50081 (*n* = 106) were collected and integrated with RNA expression data (*n* = 416) for prognostic model validation.

Metagene of 28 immune cell subtypes was obtained from https://www.cell.com/cms/10.1016/j.celrep.2016.12.019/attachment/f353dac9-4bf5-4a52-bb9a-775e74d5e968/mmc3.xlsx (Charoentong et al., 2017) to evaluate the infiltration level of immune cells by the single sample gene set enrichment (ssGSEA) method.

### Validation of the Data Grouping

ssGSEA and hierarchical cluster were used to divide the subjects into a high immune-cell-infiltrated group and a low immune-cell-infiltrated group. ESTIMATE algorithm was used to validate the grouping by comparing the stromal score, immune score, ESTIMATE score, and tumor purity of the two groups.

### Identification of Immune-Related Differentially Expressed lncRNAs

R package *edgeR* was used to find the differentially expressed genes (DEGs) between two pairs: tumor and non-tumor cells (GSE31210, GSE30219, GSE19188, and GSE27262), and high immune-infiltrated and low immune-infiltrated cells (TCGA dataset). | Log fold change| >1 and *p* < 0.05 were used to choose the DEGs because the data have been log-transformed. Then, the lncRNAs that appeared in both groups will be regarded as immune-related lncRNAs.

### Prognostic Signature Construction and Validation

The least absolute shrinkage and selection operator (LASSO) regression was used to find out the prognosis-related lncRNAs in the immune-related lncRNAs because it is a robust feature selection algorithm. The survival data of 585 TCGA patients were used. Then univariate Cox regression filtered out those prognostic lncRNAs with *p* < 0.005. Finally, stepwise multivariate Cox regression based on AIC (Akaike information criterion) value was used on the identified lncRNAs to select the ones that minimize AIC to attain the best model fit. The eventual risk score was calculated based on the coefficients of every lncRNAs as below:

(1) $$\mathrm{risk}\,\mathrm{score}=\sum_{\text{i}=1}^{\text{n}}\text{coefi}\times \text{id}$$

All the subjects were divided into high-risk and low-risk groups with respect to the median risk score. Then, the Kaplan–Meier curve was constructed to compare the overall survival (OS) between these two groups. Although the sequencing and processing methods were different for training and testing datasets, the relative gene expression level should be similar. Therefore, it is reasonable to use GEO datasets to test the prognostic lncRNA signature based on the defined coefficients. The area under the ROC curve (AUC), an evaluation of the performance of the model based on true-positive rate and false-positive rate, was plotted to assess the model. Univariate and multivariate Cox regressions were then used to explore whether the risk signature was an independent prognostic factor.

### Gene Set Enrichment Analysis (GSEA)

Hallmark gene sets were fetched from the MsigDB database using *msigdbr* (v7.0.1) package in R. The gene list was ranked by the Wald test statistics. R package *fgsea* (v1.14.0) was used to perform GSEA and visualize the top enriched gene sets.

### Pearson Correlation Analysis

Infiltration values of immune cell subtypes for LUAD were downloaded from the TIMER database^2^ (Li et al., 2016). The Pearson correlation was calculated between risk scores and infiltration value.

### Statistical Analysis

All statistical methods were accomplished by R (4.0.1) using packages *gsva, estimate, glmnet, survival*, and *fgsea*. Two-tailed *p* < 0.05 indicated significant difference if not specified.

## Results

### Gene Expression Data Grouping and Validation

**FIGURE 1:**
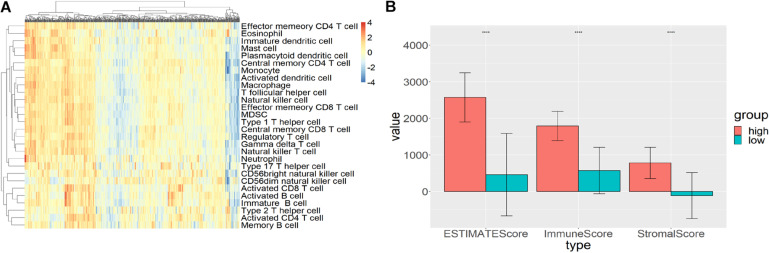
High and low immune-cell-infiltrated groups. **(A)** The GSEA scores for 28 types of immune cells from *GSVA* package using ssGSEA method. Red represented high GSEA score for high immune cell infiltration, blue represented low GSEA score for low immune cell infiltration. **(B)** The stromal score, immune score, and ESTIMATE score for high and low immune-cell-infiltrated groups.

Single sample gene set enrichment analysis (ssGSEA) and hierarchical clustering algorithm were used to divide the subjects into high immune cell infiltration (*n* = 193) and low immune cell infiltration (*n* = 392) groups. R package *GSVA* was used to calculate the GSEA score for each sample (Figure 1A). Then, the *hclust* package was used to hierarchically cluster the samples based on the Euclidean distance of these scores. The two groups derived from clustering were validated by the ESTIMATE algorithm. Compared with the high immune-cell-infiltrated group, the tumor purity of the low immune-cell-infiltrated group was significantly higher while the stromal score, immune score, and ESTIMATE score were significantly lower (*p* < 0.0001; Figure 1B).

### Identification of Immune-Related DEGs

**FIGURE 2:**
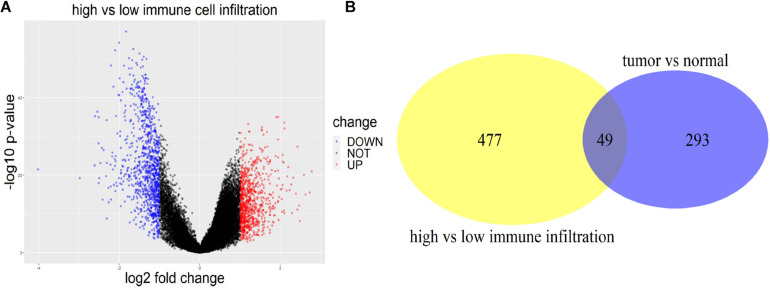
DEGs between high and low immune infiltration groups, and between tumor and normal tissues. **(A)** The volcano plot of DEGs between the high immune-cell-infiltrated and low immune-cell-infiltrated group. Red indicated DEGs up-regulated in the high infiltration group, while blue indicated the down-regulated ones. **(B)** The yellow circle represented the DE lncRNAs between high and low immune infiltration groups. The purple circle represented the DE lncRNAs between tumor and normal tissues. Forty-nine lncRNAs appeared in both groups.

R package *edgeR* was used to figure out the DEGs between tumor and normal tissues with a threshold of | log2 fold change| >1 and *p* < 0.05 using four datasets (GSE31210, GSE19188, GSE30219, and GSE27262). The DEGs were first identified within each dataset, and then the genes verified in more than one dataset were extracted. In total, 2931 DEGs including 342 lncRNAs of LUAD patients were identified for tumor and normal tissues. With the same criterion, 1,886 (874 up-regulated and 1,012 down-regulated) immune-related DEGs including 526 lncRNAs were found using TCGA data between high and low immune-cell-infiltrated groups (Figure 2A). Two-way Venn analysis was carried out to filter the immune-related DEGs for LUAD patients (Figure 2B).

### Immune-Related lncRNA Prognostic Signature Construction Using Regressions

**FIGURE 3:**
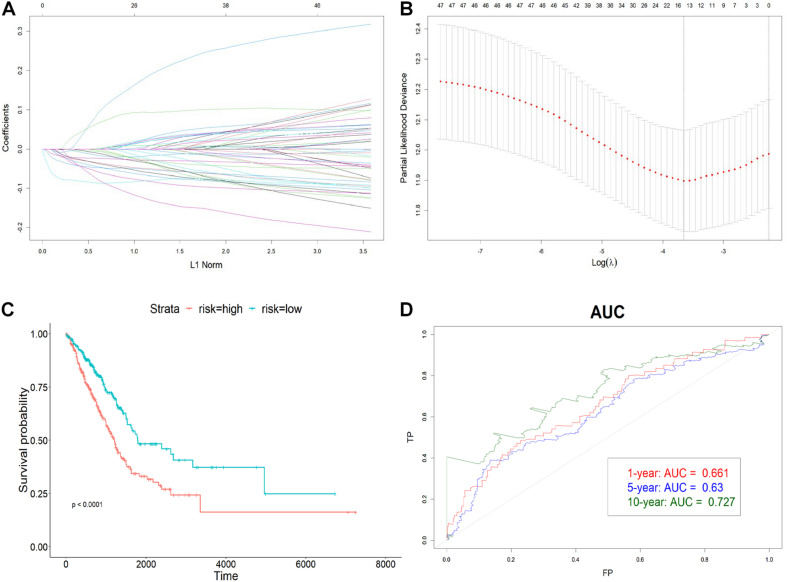
Construction of the immune-related lncRNA signature. **(A)** The LASSO coefficient profiles of 19 prognosis-related lncRNAs. Each colored line showed the change of the coefficient of one lncRNA with the normalization factor. **(B)** Partial likelihood deviance was plotted against the logarithm of lambda in the 10-fold cross-validation. The red dots indicated the deviance and the gray vertical lines indicated standard error of the deviance. The gray vertical dotted line corresponded to the optimal lambda with the lowest partial likelihood deviance. **(C)** Kaplan–Meier curve of high-risk and low-risk groups. **(D)** The AUC of 1-, 5-, and 10-year OS. The *x*-axis represented the false-positive (FP) rate, and the *y*-axis represented the true-positive (TP) rate. The signature predicted the 10-year survival best.

To avoid overfitting, 19 prognostic lncRNAs were selected from the 49 DE lncRNAs using LASSO regression with 10-fold cross-validation (Figures 3A,B). Univariate Cox regression was then carried out to increase the robustness with a threshold of *p* < 0.005 and filtered nine lncRNAs for the subsequent step. The four-lncRNA signature was finally constructed by a stepwise multivariate Cox regression with coefficients. The risk score was calculated as below:

Risk score = −0.088^∗^HSPC078 − 0.083^∗^DRAIC − 0.045^∗^AP004608.1 − 0.125^∗^MIR223HG, which is the sum of the multiplication of lncRNA expression and each coefficient.

To determine how well the risk score could predict OS, LUAD patients were divided into high-risk and low-risk groups with respect to the median risk score. The Kaplan–Meier curve showed that the OS of the high-risk group was significantly worse than that of the low-risk one (*p* < 0.0001) (Figure 3C). Also, the AUC plot suggested that the signature could predict the survival well in a long time course (0.661, 0.63, and 0.727 for 1-, 5-, and 10-year survival) (Figure 3D).

### Validation of the Effectiveness of lncRNA Signature

**FIGURE 4:**
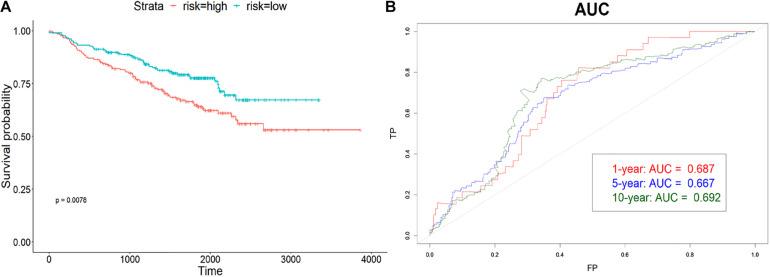
Validation of the signature. **(A)** Kaplan–Meier curve of high-risk and low-risk groups in combined validation dataset. **(B)** The AUC of 1-, 5-, and 10-year OS. The validation data also predicted 10-year survival best.

The model was validated using GSE31210, GSE30219, and GSE50081 datasets using the coefficients trained previously. Samples in each dataset were assigned to the high-risk or low-risk group based on the median risk score. Then, the assignment results of three datasets were combined to plot the Kaplan–Meier curve and AUC. From Figure 4A, the survival time of the high-risk group was significantly shorter (*p* < 0.01) than the low-risk group in the combined validating set, which suggested that the risk score can predict the OS well. The area under ROC was 0.687, 0.677, and 0.697 for the 1-, 5-, and 10-year OS (Figure 4B). Same as the training set, the lncRNA signature predicted the 10-year survival best.

### The Immune-Related Signature Could Serve as an Independent Prognostic Factor

**FIGURE 5:**
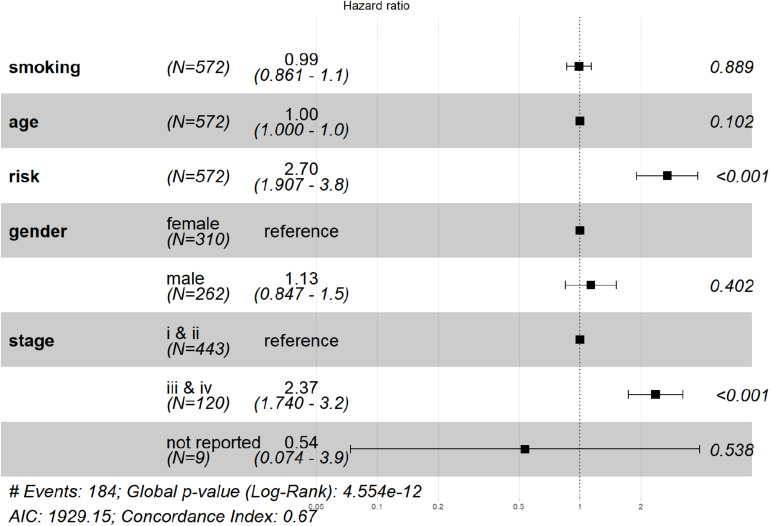
Verification that the signature is an independent prognostic factor. From left to right, the column represented: factor name, number of subjects, HR (lower and upper 95% value), the HR plot, and the *p*-value. The result of multivariate Cox regression showed that risk score and tumor stage are significant prognostic factors for LUAD patients.

The risk score was then analyzed by Cox regression along with age, gender, tumor stage, and smoking history as an independent factor. The *p* value of risk score < 0.001 in both univariate and multivariate (Figure 5). Cox regression indicated that risk score could serve as an independent prognostic factor. The risk score and advanced tumor stage were risk factors for LUAD patients with a hazard ratio (HR) larger than 1 as shown in Figure 5.

### Functional Analysis Revealed Related Signaling Pathways and Micro-RNAs

**FIGURE 6:**
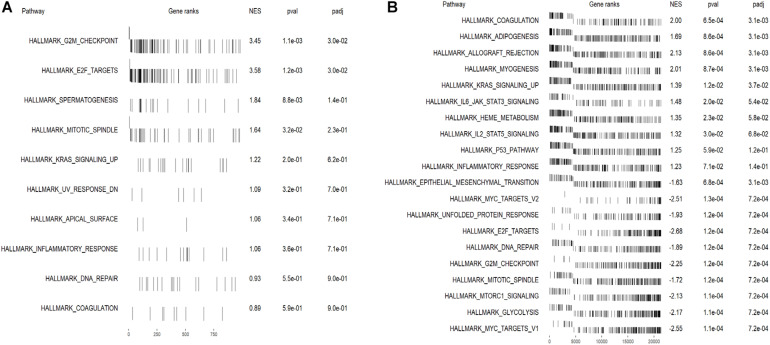
The top enriched gene sets in up- and down-regulated DEGs in the high-risk group. **(A)** Gene sets enriched in DEGs up-regulated in the high-risk group. G2M checkpoints and E2F target-related genes were significantly enriched. **(B)** Gene sets enriched in DEGs down-regulated in the high-risk group. Genes related to interleukin, STAT, KRAS, and p53 were enriched, while cell mitosis-related genes were significantly deprived.

To identify the enriched gene sets for DEGs ranked by the Wald test statistics, the *fgsea* package was used to do GSEA analysis for up- and down-regulated genes in the high-risk group separately. Several mitosis-related gene sets including E2F target (NES = 3.58) and G2M checkpoints (NES = 3.45) were enriched in the up-regulated DEGs in the high immune-cell-infiltrated group (Figure 6A). In the down-regulated DEGs, signaling pathways including IL6-JAK-STAT3, KRAS (down-regulated), IL2-STAT5, and p53 pathways were enriched (Figure 6B). These results showed that the immune-related lncRNAs may promote cancer progression by advancing cell mitosis.

Also, some micro-RNAs (miRNAs) were related to these immune-related prognostic lncRNAs. From the LncBase database (Paraskevopoulou et al., 2016), we found that 21 miRNAs have been verified to interact with these lncRNAs by experiments. The genes regulated by these miRNAs were enriched in ECM-receptor, viral carcinogenesis, p53 signaling, and hippo signaling pathways (Ioannis et al., 2015; Dimitra et al., 2018). mir-30, mir-10, and mir-181 played important roles in these pathways.

### The lncRNA Signature Was Associated With B Cell, CD4^+^ T Cell, Macrophage, and Myeloid Dendritic Cell Infiltration

**FIGURE 7:**
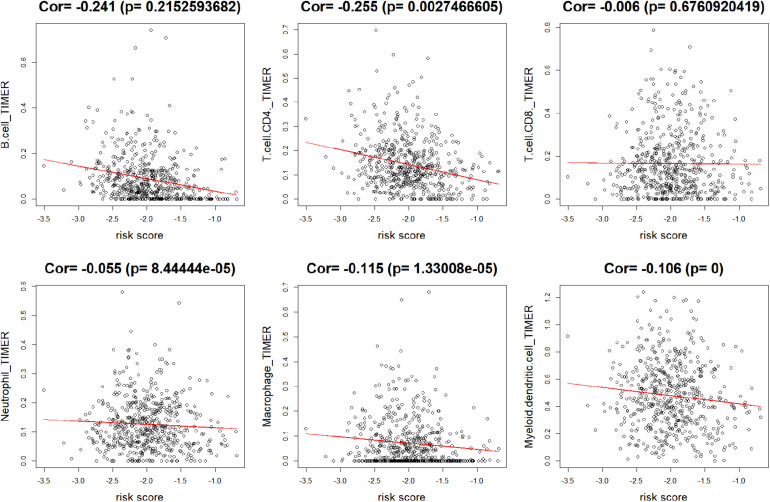
Correlation between risk score and immune cell subtype infiltration. The correlation values of B cells, CD4^+^ T cells, CD8^+^ T cells, neutrophils, macrophages, and myeloid dendritic cells were −0.241, −0.255, −0.006, −0.055, −0.115, and −0.106, respectively. Only B cells, CD4^+^ T cells, macrophages, and myeloid dendritic cells were significantly correlated with risk score (*p* < 0.05).

To explore the relationship between lncRNAs and the infiltration of some representative immune cells, the Pearson correlation value was calculated between risk scores and TIMER estimated infiltration value. As shown in Figure 7, the infiltration values of B cells, CD4^+^ T cells, macrophages, and myeloid dendritic cells were significantly negatively correlated with risk scores. The negative coefficients illustrated that the immune-related lncRNA signature was associated with high infiltration of immune cell subtypes.

## Discussion

We obtained data from TCGA and GEO database to identify immune-related differentially expressed lncRNAs of LUAD patients. Patients were grouped into high and low immune-cell-infiltrated groups by GSVA, which was further validated by ESTIMATE. LASSO regression, univariate Cox regression, and stepwise multivariate Cox regression were used to build a four-lncRNA prognostic signature. The risk score was calculated using the coefficients of the four lncRNAs, based on which patients were classified into low-risk and high-risk groups. The OS of the high-risk group was significantly shorter than the low-risk group in both the training and the testing datasets. The AUCs showed that the risk signature has a good prediction of 10-year survival. The lncRNA signature was confirmed to be an independent prognostic factor when analyzed by multivariate Cox regression along with age, gender, tumor stage, and smoking history. Finally, the functional GSEA analysis was performed to investigate how the lncRNAs may affect the OS.

Our model showed consistent results in predicting OS using both RNA-seq and microarray datasets although the coefficients were trained only by the RNA-seq data. This could be explained by the robust prognostic value of the four lncRNAs. All lncRNAs in the signature, including SIGLEC17P, DRAIC, MIR223HG, and AP004608.1, are protective for LUAD patients as shown by the negative coefficients. SIGLEC17P was suppressed in the advanced stage of cancer (iii and iv), which illustrated that the dysfunction of it may be associated with cancer progression (Zhou et al., 2019). Previous studies have implied that DRAIC may inhibit cell migration and invasion and predict longer survival time in LUAD patients (Sakurai et al., 2015). AP004608.1 was a protective lncRNA in pancreatic adenocarcinoma (Wang et al., 2019). MIR223HG has also been identified as a prognostic lncRNA related to tumor microenvironment in another study (Jin et al., 2020) with HR < 1, which was consistent with our results.

The GSEA results indicated that genes highly expressed in the high-risk group could promote cell mitosis, while genes expressed lowly seems to promote p53 IL6-JAK-STAT3 and IL2-STAT5 pathways and decrease KRAS signaling. The tumor suppressor protein p53 was suggested to regulate cell growth by promoting apoptosis and DNA repair under stressful conditions (Kanapathipillai, 2018). KRAS signaling is oncogenic and was reported to regulate tumor-associated immune responses such as inducing cancer cell evasion from immunosurveillance (Dias Carvalho et al., 2017). Therefore, the down-regulation of KRAS could delay cancer progression and benefit immunotherapy. The JAK-STAT gene set was enriched in up-regulated DEGs as a downstream pathway of interferon-gamma signaling, which is an essential responsive cytokine in cytotoxic T cells mediated killing of tumor cells (Barnholt et al., 2009; Ni and Lu, 2018). These pathways enriched in down-regulated DEGs in the high-risk group have contributed to tumor suppression in various ways that are associated with tumor immunity. Also, these lncRNAs were correlated with miRNAs including mir-30, mir-10, and mir-181. Mir-30 was shown to be a tumor suppressor gene by many studies (Braun et al., 2010; Cheng et al., 2012). As mir-10 were de-regulated in many cancers (Lund, 2010), their up-regulation may decrease the progression of cancer. The down-regulation of mir-181 was suggested to regulate PTEN expression and thus inhibit tumor development (Chang et al., 2017).

As shown by the correlation analysis, the negative correlation between risk scores and infiltration values illustrated that higher expression of lncRNA signature was correlated with higher immune cell infiltration and thus longer OS. This might be explained by the fact that the signaling pathways correlated with lncRNA expressions that could also affect[ tumor immunity.

In conclusion, we identified a novel four-lncRNA prognostic signature that was associated with the infiltration of immune cell subtypes.

## Data Availability Statement

Publicly available datasets were analyzed in this study. This data can be found here: https://portal.gdc.cancer.gov/projects/TCGA-LUAD.

## Ethics Statement

The studies involving human participants were reviewed and approved by the TCGA Ethics, Law and Policy Group. The patients/participants provided their written informed consent to participate in this study.

## Author Contributions

JW analyzed data and wrote the manuscript. XY searched manuscripts and cleaned data. XJ designed the research and modified the manuscript. Y-QZ designed the research and provided clinical insights. All authors contributed to the article and approved the submitted version.

## Conflict of Interest

The authors declare that the research was conducted in the absence of any commercial or financial relationships that could be construed as a potential conflict of interest.

## Footnotes

1 https://portal.gdc.cancer.gov/

2 http://timer.cistrome.org/
